# The Eyes Have It: A Low-Cost Model for Corneal Foreign Body Removal Training

**DOI:** 10.21980/J82S85

**Published:** 2020-01-15

**Authors:** Tabitha Ford, Megan L Fix, Troy E Madsen, Susan Stroud

**Affiliations:** * University of Utah School of Medicine, Department of Emergency Medicine, Salt Lake City, UT

## Abstract

**Audience:**

This corneal foreign body simulator is designed to instruct junior emergency medicine (EM) residents and medical students with an interest in emergency medicine.

**Introduction:**

Eye complaints are common in the emergency department (ED), accounting for approximately 2 million ED visits each year.^1^ Corneal foreign bodies (CFB) account for approximately 7.5% of these presentations, and many EM providers are uncomfortable with removal procedures.^1^–^3^ Simulation has been demonstrated to improve provider comfort with this skill.^4^,^5^ Previous models for CFB removal have been created using wax over glass spheres, molded materials with silicone and ballistics gel, bovine eyes, cardboard glove boxes with ink stains simulating foreign bodies and rust rings, and agar plates with pepper-corns. 4–9 Often, these models are expensive or time-consuming to create or lack spatial realism.

We propose that a simple, inexpensive model will be effective in increasing emergency provider comfort with CFB removal under slit lamp magnification in addition to increasing provider comfort using a slit lamp.

**Educational Objectives:**

By the end of the session, the learner should be able to adequately focus a slit lamp in order to identify and magnify a corneal foreign body and demonstrate safe technique for removal of a corneal foreign body under slit lamp guidance.

**Educational Methods:**

We created a low-fidelity CFB simulator for approximately $15 utilizing a Styrofoam ball, toothpicks, grapes, novelty glasses, and magnesium shavings. Toothpicks secured grapes into simulated orbits, scooped out of a Styrofoam ball. We fastened the Styrofoam ball to the slit lamp using medical tape. We added novelty glasses to simulate working around facial features. A senior resident instructor then used forceps to insert small magnesium shavings into the grapes to simulate foreign bodies. Participants received an introduction on techniques for successful CFB removal using the bevel of a needle under slit lamp guidance.^10^,^11^ They practiced using the models under supervision of an instructor.

**Research Methods:**

We conducted a prospective trial using a convenience sample of 19 learners at a university-based EM residency program, including EM interns, one emergency advanced-practice clinician, and fourth-year medical students participating in an EM sub-internship. We analyzed results using a Fisher’s exact test.

**Results:**

Before training, few participants (36.8%) had observed a corneal foreign body removal, and only 15.8% had performed the procedure. More than half (52.6%) of participants said they were somewhat or very comfortable using a slit lamp before the training and 89.5% were somewhat or very comfortable after training (p=0.029). None of the participants were somewhat or very comfortable removing CFBs before the training and 84.2% were somewhat or very comfortable post-training (p<0.001).

**Discussion:**

Results suggest that simulation with this low-cost model effectively improves provider comfort in CFB removal in addition to improving comfort using a slit lamp.

**Topics:**

Eye exam, eye injury, ocular injury, corneal injury, corneal foreign body, slit lamp, corneal foreign body removal.

## USER GUIDE

[Table t2-jetem-5-1-i10]

**Table t2-jetem-5-1-i10:** 

List of Resources:	
Abstract	11
User Guide	12

**Learner Audience:**

Medical Students, Interns, Junior Residents, Senior Residents, EM APC

**Time Required for Implementation:**

**Instructor:** 30 minutes

**Learners:** approximately 45 minutes per session

**Recommended Number of Learners per Instructor:**

A single instructor can adequately supervise two groups of participants taking turns practicing on two slit lamps. Ideal groups include approximately 4–5 learners per slit lamp.

**Topics:**

Eye exam, eye injury, ocular injury, corneal injury, corneal foreign body, slit lamp, corneal foreign body removal.

**Objectives:** By the end of the session, the learner should be able to:

1. Adequately focus a slit lamp in order to identify and magnify a corneal foreign body
2. Demonstrate safe technique for removal of a corneal foreign body under slit lamp guidance

### Linked objectives and methods

This format allows learners to practice this high-stakes skill in a safe learning environment. They begin to develop visuospatial familiarity executing procedures with microscopic guidance (objective 1) while also developing the dexterity necessary to perform corneal foreign body removal on patients, all under the supervision of an instructor (objective 2). We strived to create a simulator that was inexpensive, simple, and reusable, while also providing enough realism to improve provider comfort with the skill.

### Recommended pre-reading for instructor

- We recommend that instructors brush up on their corneal foreign body knowledge by reviewing this article and watching these embedded videos:
- Nickson C. Something in my eye, Doc. Life in the Fastlane. https://litfl.com/something-in-my-eye-doc/. Updated March 4, 2019. Accessed July 25, 2019.

### Learner responsible content (LRC)

- We recommend that leaners review basic slit lamp skills by watching this video before attending the simulation session:
- Root T. Slit lamp exam (video). Tim Root:Virtual Eye Professor. https://timroot.com/slit-lamp-exam-video/. Published 2011. Accessed July 26, 2019.

### Implementation Methods

Learners unfamiliar with slit lamp basics received a brief introduction and practice session with the slit lamp before beginning the corneal foreign body simulation. We then spent 15 minutes discussing corneal foreign body removal techniques including:

- Removal attempts with irrigation or a moist cotton swab
- Using bilateral topical anesthetics to reduce patient blinking during removal
- Needle size selection
- Technique of bending the needle tip before attempting the procedure^9^
- Advantages/disadvantages of using the dominant versus non-dominant hand for the removal procedure
- Focusing the slit lamp on the CFB before picking up the needle
- Approaching the patient’s eye with the needle from the lateral aspect, then using the slit lamp magnification only after needle is in place approximately 5mm anterior to the surface of the cornea.
- Needle positioning and movements necessary to remove the CFB

After this discussion, we allowed up to 30 minutes of supervised practice by participants. Groups typically consist of 3 to 10 participants with one slit lamp available for each group of 5 learners.

### List of items required to replicate this innovation

1. 6” diameter Styrofoam ball- $5.50/ball - https://www.amazon.com/gp/product/B0721S63QL/ref=ppx_yo_dt_b_asin_title_o09_s00?ie=UTF8&psc=1
2. Novelty glasses (flexible, with no lenses, and with a false nose)- $1.72/pair - https://www.amazon.com/gp/product/B005AW35NM/ref=ppx_yo_dt_b_asin_title_o09_s02?ie=UTF8&psc=1
3. Toothpicks-$1/box - local grocery store
4. Red seedless grapes- $2/lbs - local grocery store
5. Magnesium fire starter- $4.99/block - https://www.amazon.com/HTS-222B0-Magnesium-Starter-Striker/dp/B00UXVFC72/ref=sr_1_1?keywords=magnesium+fire+starters&qid=1564107088&refinements=p_85%3A2470955011&rnid=2470954011&rps=1&s=gateway&sr=8-1

### Approximate cost of items to create this innovation

Each model can be constructed for approximately $15.

### Detailed methods to construct this innovation

1. Place the novelty glasses on the Styrofoam ball and mark the location of the eyes. Use a knife/spoon to scoop out orbits in a size just larger than the grapes. Allow room for the grapes to protrude from the sockets.
2. Insert one toothpick into each orbit, leaving approximately 1cm of the toothpick exposed.
3. Tape the novelty glasses into place.
4. Draw or decorate the remainder of face as desired.[Fig f1-jetem-5-1-i10]
5. Insert metal magnesium shavings into the surface of the grape eyes using forceps or an 18g needle (which participants may use later for foreign body removal).[Fig f2-jetem-5-1-i10]
6. Insert grapes into orbits, skewering one grape on each toothpick so that foreign bodies are exposed anteriorly.[Fig f3-jetem-5-1-i10]

### Results and tips for successful implementation

This technique has been used on multiple occasions at our academic facility with consistent positive learner feedback: during intern education, medical student rotations, and an annual didactics week for senior residents. During the first two sessions, under Institutional Review Board approval, we administered pre- and post- simulation surveys, measuring learner comfort with slit lamps and CFB removal. Results suggest that these sessions improve provider comfort with both slit lamp manipulation and CFB removal.

We have also received feedback that skills acquired during training with this model translate well into successful subsequent attempts at CFB removal on human patients.[Table t1-jetem-5-1-i10]

All pieces of the simulator are reusable and easily stored for future use with the exception of the grapes. We have learned that red grapes tend to have improved structural integrity for this simulation compared to green grapes. In addition, alternative metallic shrapnel has been created with small fragments of a staple (cut with trauma shears while wearing eye protection) or shavings from a paper clip. However, we have found magnesium shavings to be the least time/labor intensive to produce.

## Figures and Tables

**Figure 1 f1-jetem-5-1-i10:**
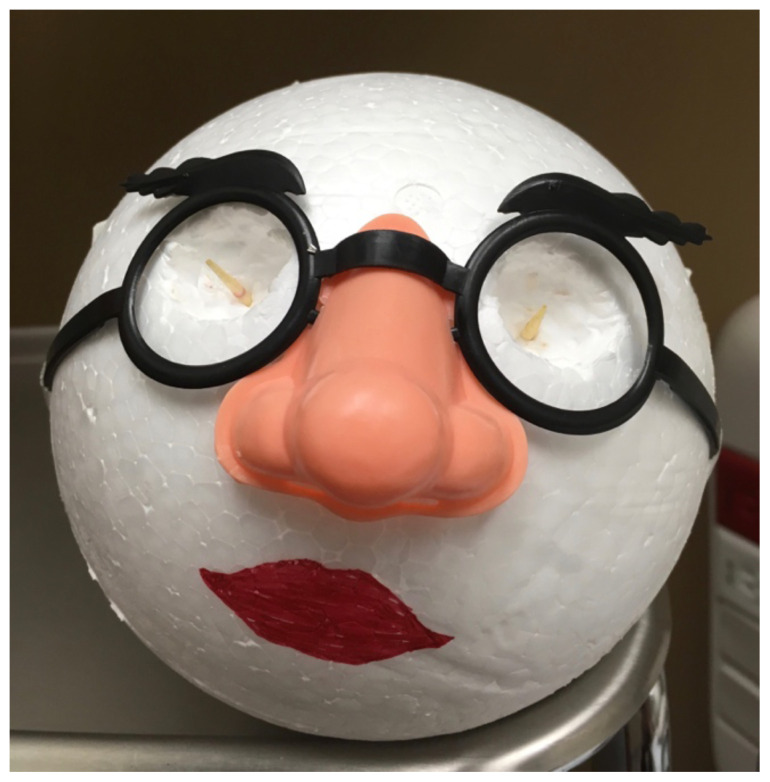
Model before insertion of grapes: Author’s own image.

**Figure 2 f2-jetem-5-1-i10:**
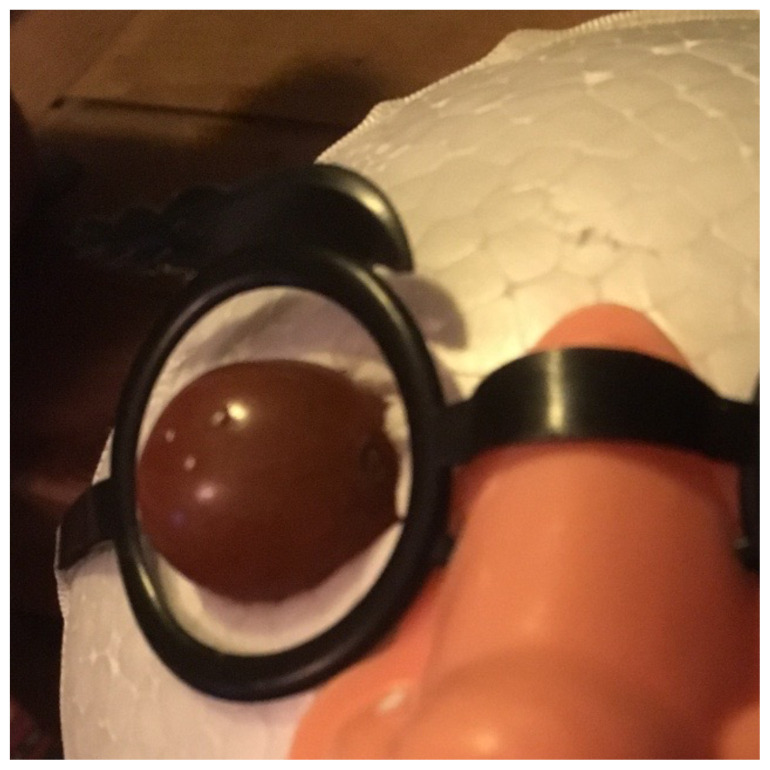
Metallic foreign bodies in place: Author’s own image.

**Figure 3 f3-jetem-5-1-i10:**
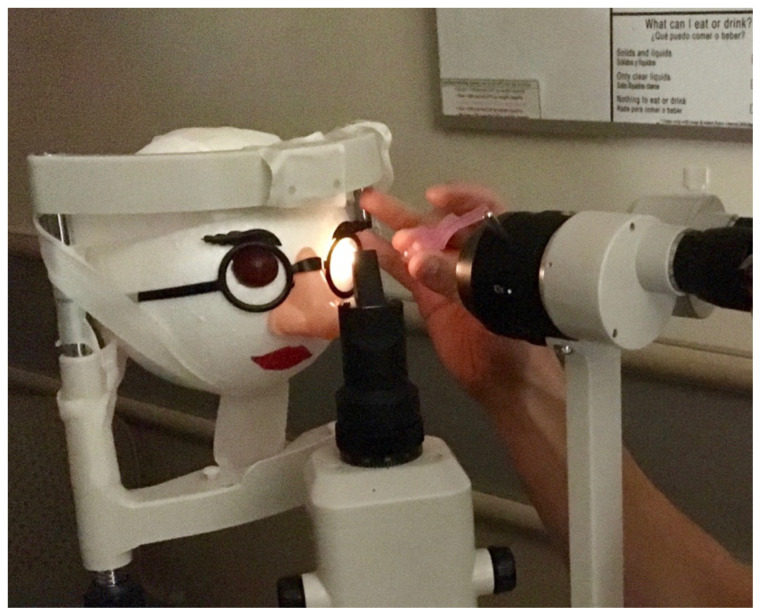
Completed model in use: Author’s own image.

**Table 1 t1-jetem-5-1-i10:**
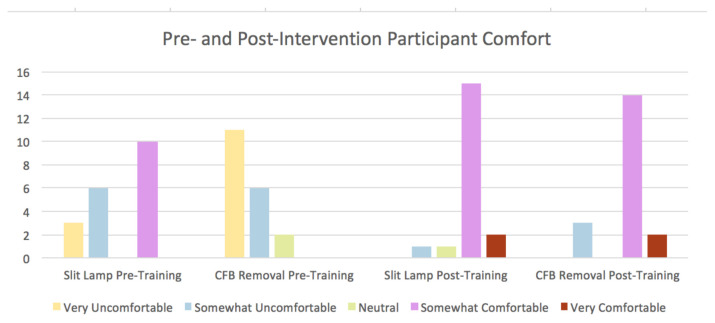
Effects of training on participant comfort with slit lamp manipulation and corneal foreign body removal: Author’s own image.
